# Polyphenol-Retaining Decaffeinated Cocoa Powder Obtained by Supercritical Carbon Dioxide Extraction and Its Antioxidant Activity

**DOI:** 10.3390/foods2040462

**Published:** 2013-10-14

**Authors:** Kinji Kobori, Yuto Maruta, Shigeru Mineo, Toru Shigematsu, Masao Hirayama

**Affiliations:** 1 Bourbon Institutes of Health, Bourbon Corporation, 4-2-14 Matsunami, Kashiwazaki City, Niigata 945-8611, Japan; E-Mails: maruta-yut@bourbon.co.jp (Y.M.); mineo-shi@bourbon.co.jp (S.M.); hirayama@nbrp.co.jp (M.H.); 2 Faculty of Applied Life Sciences, Niigata University of Pharmacy and Applied Life Sciences, 265-1 Higashijima, Akiha-ku, Niigata City, Niigata 956-8603, Japan; E-Mail: shige@nupals.ac.jp

**Keywords:** cocoa powder, decaffeination, supercritical carbon dioxide, antioxidant activity

## Abstract

Cocoa beans contain many functional ingredients such as theobromine and polyphenols, but also contain a relatively high amount of caffeine, which can negatively impact human health. It is therefore desirable to reduce caffeine levels in cocoa powder used to make chocolate or cocoa beverages while retaining functional ingredients. We have established conditions for supercritical carbon dioxide (SCCO_2_) extraction that remove 80.1% of the caffeine from cocoa powder while retaining theobromine (94.1%) and polyphenols (84.7%). The antioxidant activity of the decaffeinated cocoa powder (DCP) made with this optimized SCCO_2_ extraction method was 85.3% that of non-processed cocoa powder. The total procyanidin and total polyphenol concentrations of the DCPs resulting from various SCCO_2_ extractions showed a significant positive correlation with oxygen radical absorbance capacity (ORAC). The correlation coefficient between total polyphenols and ORAC was higher than that between total procyanidins and ORAC; thus, the concentration of total polyphenols might be a greater factor in the antioxidant activity of DCP. These results indicate that we could remove large quantities of caffeine from conventional high-cocoa products while retaining the functional benefits of high polyphenol content. This SCCO_2_ extraction method is expected to be applicable high-cocoa products, such as dark chocolate.

## 1. Introduction

Cocoa powder, chocolate, and various other products made from cocoa beans (*Theobroma cacao* L.) are health-benefitting foods containing various functional ingredients. Cocoa powder contains methylxanthines, such as theobromine and caffeine. The unfavorable stimulation of theobromine is weaker than that of caffeine, and theobromine has been reported to lower blood pressure [1] and increase plasma HDL-cholesterol concentration [2]. The polyphenols in cocoa such as catechins and procyanidins (monomers through polymers) have shown high antioxidant activity [3], as well as the ability to improve blood pressure and blood vessel function [4]. Furthermore, polyphenols have been shown to improve insulin resistance and glucose tolerance [5] as well as platelet function [6], inhibiting inflammation and allergic inflammation [7], suppressing the onset of cancer [8], enhancing positive mood [9], and there is possibly preventing dementia [10]. It has been confirmed that consuming chocolate, which includes cocoa mass, leads to decreased risk of cardiovascular disease [11] and stroke [12]. Another methylxanthine and structural isomer of theobromine, caffeine, is also found in cocoa beans. It has been reported that caffeine decreases risk of cardiovascular disease [13] and depression among women [14], and Parkinson’s disease [15] in a moderate intake. On the other hand, it has also been reported to acutely increase blood pressure [16,17] and increase alertness and excitatory action through increasing of plasma epinephrine [17]. However, caffeine’s stimulating effects are stronger than theobromines, and overconsumption or overdose can cause insomnia [18], increase risk of infertility in women [19], cause spontaneous abortion [20], and restrict fetal growth [21]. Thus, cocoa powder contains ingredients that are both beneficial and harmful to human health. When tracking caffeine intake, it is necessary to note overall consumption from all dietary sources. Many people regularly consume chocolate and cocoa drinks because of their high palatability, and high-cocoa products such as dark chocolate are often consumed for their health benefits, but an excess of caffeine may be consumed alongside the desired functional ingredients. Depending on the quantity consumed, consumption of milk chocolate does not result in excessive intake of caffeine. However, on the other hand, consumption of dark chocolate may result in the same caffeine intake as one cup of coffee [22]. Therefore, in order to obtain sufficient functionality of cocoa products, it is necessary to selectively remove as much caffeine as possible from cocoa powder while maximizing theobromine and polyphenol retention. 

Supercritical carbon dioxide (SCCO_2_) extraction has been used industrially to produce decaffeinated products by removing caffeine from coffee beans and green tea. This process involves separating one ingredient from a sample matrix using a supercritical fluid as the extracting solvent. Extraction conditions for SCCO_2_ are above the critical temperature and pressure for carbon dioxide, 31.1 °C and 7.38 MPa, and the resulting supercritical fluid is an efficient solvent for nonpolar solutes such as oils and fats. The solvent power of SCCO_2_ can be altered by adding cosolvents such as water or ethanol, or by varying the pressure and temperature, which allows for selective extraction of specific ingredients from the matrix. 

It has been reported that extraction of xanthine (theobromine and caffeine) is possible from a non-roasted, raw cacao nib [23]. However, decaffeination of cocoa beans in an industrial setting has hardly been practiced compared with that of coffee beans. One reason is that the lipid content of cocoa beans is high, about 55%. Lipids, which are nonpolar, are extracted in preference to other ingredients because SCCO_2_ is also nonpolar. Therefore, the decaffeination of cocoa beans is expected to be much less efficient than that of coffee beans or tea leaves, which have lower lipid content. In Park *et al*.’s study [24] of caffeine extraction from tea leaves by SCCO_2_, under extraction conditions of a fixed temperature of 70 °C, a fixed pressure of 30 MPa, a fixed solvent-to-feed ratio of 102.0 wt% (S/F ratio: SCCO_2_ weight (g)/sample weight (g)), and a fixed cosolvent (water) addition ratio of 897.6 wt% (cosolvent weight (g)/sample weight (g) × 100), the removal ratio of caffeine was 75.1%. In studies by Rahoma *et al.* [25] on caffeine and theobromine extraction from cocoa beans, under SCCO_2_ extraction conditions of a fixed temperature of 70 °C, a fixed pressure of 40 MPa, and a fixed S/F ratio of 433.3, the removal ratios of caffeine and theobromine were 66.4% and 29.9%, respectively. However, the extraction time for cocoa beans might be longer than that for tea leaves because, in the above studies, the S/F ratio for cocoa beans was about 4.2 times higher. Therefore, the efficiency of caffeine extraction from cocoa beans was lower than that of tea or coffee. In addition, the post-extraction theobromine content was remarkably lower. Furthermore, the valuable polyphenol content of cocoa beans would decrease greatly under such high-temperature, high-pressure, long SCCO_2_ extraction conditions, and cocoa beans’ polyphenol-derived functionality would be expected to decrease. For example, in Park *et al.*’s [24] examination of tea leaves, epigallocatechin gallate (EGCG) was greatly reduced, with a 69.0% removal ratio. Thus, concerns remain regarding both decaffeination inefficiency and polyphenol loss.

Therefore, in this study, we aimed for the effective decaffeination of cocoa powder by SCCO_2_ extraction. Furthermore, we examined various methods of SCCO_2_ extraction in order to maximize retention of theobromine and polyphenols in the residual substance of cocoa powder following SCCO_2_ extraction. Lastly, we evaluated the functionality of each DCP provided in various SCCO_2_ extraction conditions, using oxygen radical absorbance capacity (ORAC) values as an index of antioxidant activity.

## 2. Experimental Section

### 2.1. Sample Conditions and SCCO_2_ Extraction

Cocoa beans cultivated in Ghana were purchased from Itochu Food Sales and Marketing (Japan). The beans were roasted and ground into cocoa mass. For all experiments, we used non-alkalized cocoa powder made of this cocoa mass, defatted to 16.4% from 55.1% by mechanical pressing and milled to a mean particle size of 32.7 μm.

The supercritical fluid extraction system (model SFX1220, Teledyne ISCO, USA) was used to perform SCCO_2_ extraction. First, 0.5 g of cocoa powder was placed in a cartridge for extraction, water was added as a cosolvent in proportions of 0, 15, 30, 45, 60, and 75 wt% for sample weight, and then sample and cosolvent were mixed. This cartridge was loaded in the extraction tank of the system and moistened for 10 min at temperatures of 50, 70, or 90 °C. Subsequently, under the same temperature conditions, SCCO_2_ was poured into the extraction tank and pressure was raised to 10, 20, or 30 MPa and maintained for 20 min. Under the same pressure, SCCO_2_ was continuously poured into the extraction tank at a flow rate of 3.0–4.0 mL/min until the S/F ratio reached 55.6, which is the end point for the extraction. The substance remaining after the extraction was DCP.

### 2.2. Analysis of SCCO_2_ Extraction Products

#### 2.2.1. Determination of Caffeine and Theobromine Content

We used caffeine monohydrate (98.5% purity) and theobromine (99.0%) as standards, and used benzotriazole (97.0%) as an internal standard (IS) for high-performance liquid chromatography (HPLC). All the standards were purchased from Kanto Chemical (Japan). A 0.2 g portion of the freeze-dried sample was mixed with 1.0 mL of IS solution (10 mg/mL). The volume was adjusted to 20 mL by adding distilled water, and the solution was heated at 100 °C for 30 min. After water-cooling for 10 min, distilled water was added again to bring the final volume to 20 mL. This solution was diluted10-fold and centrifuged at 3000 rpm for 5 min. Then the supernatant was filtered with a 0.45 μm polypropylene membrane syringe filter and the resulting solution was used for determining the caffeine and theobromine content. The caffeine and theobromine (methylxanthines) content was determined by HPLC using a Prominence Chromatograph (Shimadzu Corp., Japan) with an Inertsil ODS-3 column (GL Sciences, Japan). Isocratic elution of 80% 0.01 M phosphate buffer and 20% acetonitrile was used at a flow rate of 1 mL/min. Methylxanthines were identified using a UV detector at 275 nm, and quantified using a calibration curve prepared by the internal standard method [26].

#### 2.2.2. Determination of Total Polyphenol Content

Folin-Ciocalteau phenol reagent, (+)-epicatechin (used as a standard), and Na_2_CO_3_ were purchased from Sigma-Aldrich Japan (Japan), Sigma-Aldrich (USA), and Kanto Chemical (Japan), respectively. A 0.2 g portion of the untreated freeze-dried cocoa powder and DCP were mixed with 800 μL of distilled water. This solution was placed in 70 °C water for 5 min. Four milliliters of hexane was then added to the solution, which was subsequently shaken for 10 min and then centrifuged at 3000 rpm for 5 min with the hexane layer removed for defatting. Twelve milliliters of 50% ethanol was added to the solution, which was subject to ultrasonic extraction at 70 °C. Then 50% ethanol was then added to bring the solution to 20 mL, the resulting mixture was centrifuged at 3000 rpm for 5 min, and the supernatant was filtered with 150 mm filter paper to achieve the sample solution. This sample solution was used to determine total polyphenol and procyanidin content as well as ORAC value.

The total polyphenol content of was quantified according to the Folin-Ciocalteau method [27]. (+)-Epicatechin was dissolved in distilled water at room temperature. Fifty microliters of each sample solution or standard was mixed with 3950 μL of distilled water and 500 μL of Folin-Ciocalteau reagent. The mixture was vortexed and left to stand for 1 min at room temperature, after which 500 μL of 20% Na_2_CO_3_ was added for color stabilization. The mixture was vortexed again and left to stand for 60 min. The developed color of the mixture was then analyzed at 765 nm using a model U-1900 Hitachi ratio beam spectrophotometer (Japan). Results of total polyphenol content measurement were expressed as epicatechin equivalent values (mean ± SE).

#### 2.2.3. Determination of Procyanidin Content

The total procyanidin content was quantified according to previously reported methods [28,29]. The sample solution obtained in the above step and diluted with 50% ethanol was used to determinate procyanidin content. Chromatographic separation was performed on an HPLC fluorescence system consisting of a binary pump, solvent degasser, column oven, autosampler, system controller, and RF-10A XL fluorescence detector (Shimadzu Corp., Japan). The analysis was carried out on a Develosil 100-Diol-5 column (4.6 × 250 mm) with a particle size of 5 μm (Nomura Chemical, Japan) at a column temperature of 35 °C and injection volume of 5 μL. The binary mobile phase consisted of (A) acetonitrile and acetate (98:2 by volume), and (B) methanol, water, and acetate (95:3:2 by volume). Separations were effected by a series of linear gradients of B into A at a flow rate of 1 mL/min: 0–60 min, 0%–37.6% B in A; 60 min, 100% B in A; 60–70 min, 100% B in A; 70 min, 0% B in A; 70–80 min, 0% B in A. Fluorescence detection was recorded at an excitation wavelength of 230 nm and an emission wavelength of 321 nm. Results of the procyanidin content measurement were expressed as epicatechin equivalent values (mean ± SE).

#### 2.2.4. Calculation of the Removal Ratio and the Residual Ratio of Each Ingredient

The removal ratio and the residual ratio of each ingredient after SCCO_2_ extraction were calculated by the following equations.

Y (%) = (A − B)/A × 100(1)

X (%) = 100 − Y(2)

Here, Y and X signify the removal ratio and the residual ratio, respectively. A and B respectively express ingredient content before SCCO_2_ extraction (ingredient concentration before SCCO_2_ extraction × cocoa powder weight before SCCO_2_ extraction) and ingredient content after SCCO_2_ extraction (ingredient concentration after SCCO_2_ extraction × cocoa powder weight after SCCO_2_ extraction). The proportion of caffeine extracted by SCCO_2_ was given by the removal ratio, and the theobromine, polyphenol, and procyanidin proportions retained after SCCO_2_ extraction were given by the residual ratio.

#### 2.2.5. Evaluation of Antioxidant Activity

The sample solution 50% diluted by ethanol was used to determine ORAC. The ORAC assays were carried out for each sample solution using a Spectra Max Gemini (Molecular Devices, USA) with an excitation wavelength of 485 nm and an emission wavelength of 525 nm at an incubator temperature of 37 °C. Procedures were based on the modified ORAC method [30,31,32]. Results were expressed as micromoles of Trolox equivalents per gram (μmol-TE/g).

### 2.3. Statistical Analysis

All samples were assayed at least in triplicate in each examination and results expressed as mean ± SE. Statistical analysis was conducted using Statcel 2 software (OMS Publishing, Japan). The correlation of total polyphenol or procyanidin concentration with ORAC was analyzed using a linear regression of data calculating the coefficient of the Pearson correlation (*r*) at a 95% confidence level. Statistical significance was judged when the two sided *p*-value was less than 0.05 (*p* < 0.05).

## 3. Result and Discussion

### 3.1. Composition of Cocoa Powder before SCCO_2_ Extraction

Table 1 shows the nutritional information, as well as caffeine, theobromine, total polyphenol, and total procyanidin concentrations, of the cocoa mass and the defatted cocoa powder used in this study. Cocoa mass concentrations were as follows: caffeine 1.19 mg/g, theobromine 15.17 mg/g, total polyphenols 33.15 mg/g, and total procyanidins 10.03 mg/g. In previous reports, cocoa mass cultivated in Ghana had the following chemical concentrations: caffeine 1.37–1.59 mg/g [33], theobromine 12.3–17.3 mg/g [33], and total polyphenols 29.3 mg/g [34]. In addition, cocoa mass made from beans cultivated in the Ivory Coast had a total procyanidin concentration of 8.6 mg/g and a fat content of 53.9% [35]. The production center and fermentation state of the cocoa beans may have influenced compound levels, but the concentrations of each ingredient reported here indicate that the cocoa mass used in this study was similar to that in previous studies. 

The cocoa powder used for SCCO_2_ extraction was defatted to 16.4%. This cocoa powder was recorded as having a caffeine concentration of 2.15 mg/g, theobromine of 29.21 mg/g, total polyphenols of 62.32 mg/g, and total procyanidins of 14.20 mg/g. The major source of polyphenols in the cocoa powder was thought to be procyanidins, as the total procyanidin content accounted for 24.3% of the total polyphenol content. The quantity of each ingredient was concentrated by defatting and no significant loss of theobromine or polyphenols was observed. For comparison, the total polyphenol and total procyanidin concentrations of cocoa powder after removal of fat were 72.3 mg/g and 17.6 mg/g, respectively.

**Table 1:** Nutritional information; caffeine, theobromine, polyphenol, and procyanidin concentrations; and oxygen radical absorbance capacity (ORAC) value of cocoa powder.

	Content (g/100 g)						Calorie (kcal/100 g)	Concentration (mg/g)				ORAC (μmol-TE/g)
	Protein	Fat	Carbohydrate	Dietary fiber	Moisture	Mineral		Caffeine	Theobromine	Total polyphenols	Total procyanidins	
Cocoa mass	14.2	55.1	25.7	15.6	1.8	3.2	655.5	1.19 ± 0.02	15.17 ± 0.03	33.15 ± 0.73	10.03 ± 0.16	NT
Cocoa powder	27.7	16.4	47.6	29.5	2.6	5.7	449.0	2.15 ± 0.01	29.21 ± 0.13	62.32 ± 1.26	15.14 ± 0.26	1428.9 ± 11.1

NT: not tested.

### 3.2. Effect of Water as a Cosolvent on SCCO_2_ Extraction

SCCO_2_ is a low-polarity or nonpolar solvent, and therefore more readily extracts similarly low-polarity or nonpolar solutes. The extraction of solutes with other given polarities is enabled by precise manipulation of SCCO_2_’s polarity using cosolvents. For the extraction of caffeine, which is highly polar, cosolvents such as water, ethanol, methanol, acetone, ether, ethyl acetate, or hexane are required to increase the polarity of SCCO_2_. Using organic solvents other than ethanol in this process is inappropriate because of potential human health risks. The need to add water to effect the extraction of xanthines is well known. For example, the commercial extraction of caffeine from coffee beans is done with the addition of 30% water using SCCO_2_ at pressure from 16 to 22 MPa. Margolis *et al*. [23] claimed that it was possible to extract the xanthines (theobromine and caffeine) from raw, unroasted cocoa nibs when the humidity was increased to 30% or 40% and using SCCO_2_ at temperatures higher than 80 °C and 30 MPa. In addition, according to previous reports, ethanol was a more effective cosolvent than water in SCCO_2_ extraction of caffeine and EGCG from tea leaves [24,36]. However, these reports also show that the use of water rather than ethanol as a cosolvent results in a higher residual ratio of polyphenols. Here we aim to retain a high quantity of beneficial compounds, including polyphenols, so water was used as a cosolvent instead of ethanol.

**Figure 1:**
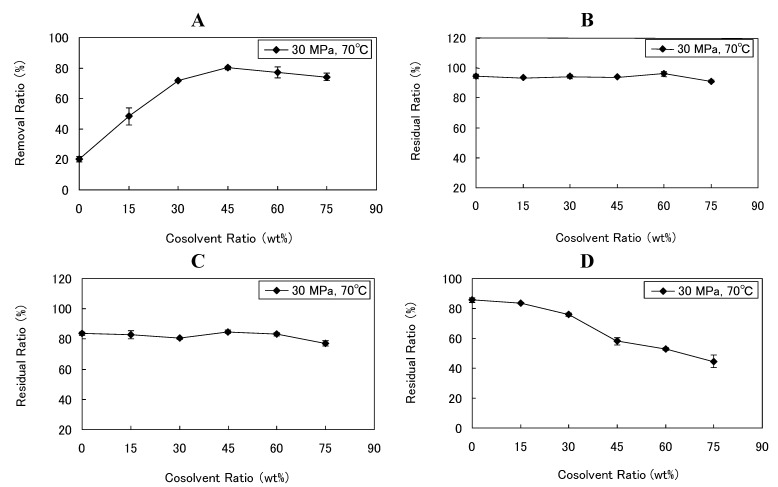
Effects of cosolvent ratio on (**A**) caffeine removal ratio and (**B**) theobromine, (**C**) total polyphenols, and (**D**) procyanidin residual ratios at a fixed pressure of 30 MPa, a fixed temperature of 70 °C, and a fixed S/F ratio of 55.6.

The content of each ingredient was quantified in the DCP produced by the following SCCO_2_ extraction conditions: a fixed temperature of 70 °C, a fixed pressure of 30 MPa, and varying quantities of cosolvent. The removal ratio of caffeine rose with the addition of cosolvent, and reached a high of 80.12% when the cosolvent ratio was 45 wt%. In addition, the trend was observed that the removal ratio of caffeine plateaued above a cosolvent ratio of 45 wt% (Table 2, Figure 1A). Water acts to free the caffeine from its bonded form in the plant matrix by hydrolysis [37]. It was thought few changes in the solubility of SCCO_2_ because the solubility of water in SCCO_2_ is very low, less than 0.01 mole fraction [38]. However, Kim *et al*. [39] reported that the solubility parameter rises as the quantity of water used as cosolvent increases, and the removal ratio of caffeine and EGCG from tea leaves likewise increases as near-total solubility is achieved [36]. Therefore, a large quantity of caffeine could be extracted because the high quantity of initial water added to the cocoa powder would free the caffeine from the plant matrix before SCCO_2_ extraction and because the water as cosolvent would raise the solubility of SCCO_2_.

The residual ratio of theobromine varied within a range of 90.98%–96.02% (Table 2, Figure 1B). This residual ratio reached its lower value when the cosolvent ratio was 75 wt%, and the quantity of cosolvent appeared to exert a small influence. It has been reported that the solubility of theobromine in SCCO_2_ increased by addition of methanol as a cosolvent, which resulted in a decreased residual ratio of theobromine [40]. Similarly, it was confirmed that the residual ratio of theobromine decreased by adding a certain quantity of the water as cosolvent in this study. The quantity of theobromine extracted at a cosolvent ratio of 75 wt% was 2.64 mg. This quantity exceeded the maximum quantity of caffeine extracted (1.72 mg) under the same SCCO_2_ extraction conditions. As the theobromine content in cocoa powder is 13.5 times higher than the caffeine content, the quantity of theobromine extracted can be greater than that of caffeine even if the removal ratio of theobromine is much lower. It has been reported that the solubility of the caffeine in SCCO_2_ is 2 orders of magnitude higher than that of theobromine [40,41]. However, the solubility of the caffeine in SCCO_2_ observed in this study was 1 order of magnitude lower than that of theobromine. The solubility of theobromine was close to the reference value, but the solubility of caffeine was much smaller than the reference value [41]. This result suggests that the value of the solubility of caffeine observed in this study was not achieved in equilibrium.

**Table 2:** Concentration and removal ratio of caffeine, concentration and residual ratio of theobromine, total polyphenols, and total procyanidins, and ORAC values in dry decaffeinated cocoa powders (DCPs) obtained by SCCO_2_ extraction with various cosolvent ratios.

SCCO_2_ extraction conditions			Concentration (mg/g)				Removal ratio (%)	Residual ratio (%)			ORAC (μmol-TE/g)
Pressure (MPa)	Temperature (°C)	Cosolvent (wt%)	Caffeine	Theobromine	Total polyphenols	Total procyanidins	Caffeine	Theobromine	Total polyphenols	Total procyanidins	
30	70	0	2.13 ± 0.05	34.24 ± 0.42	64.61 ± 0.68	12.96 ± 0.24	20.07 ± 1.79	94.56 ± 1.37	83.63 ± 1.06	85.58 ± 1.60	1287.4 ± 26.3
30	70	15	1.43 ± 0.12	35.08 ± 0.65	66.36 ± 0.79	12.64 ± 0.04	48.26 ± 5.47	93.52 ± 0.16	82.98 ± 2.67	83.47 ± 0.26	1335.3 ± 42.9
30	70	30	0.74 ± 0.01	33.50 ± 0.28	60.97 ± 0.77	11.49 ± 0.16	71.89 ± 0.15	94.32 ± 1.12	80.46 ± 0.75	75.92 ± 1.04	1203.0 ± 53.1
30	70	45	0.52 ± 0.09	33.44 ± 0.08	64.17 ± 0.98	8.79 ± 0.37	80.12 ± 1.05	94.12 ± 0.02	84.67 ± 1.13	58.06 ± 2.43	1218.5 ± 50.0
30	70	60	0.58 ± 0.09	33.00 ± 0.51	60.21 ± 0.73	7.99 ± 0.15	77.10 ± 3.55	96.02 ± 1.49	83.12 ± 1.00	52.76 ± 0.97	1203.8 ± 14.6
30	70	75	0.66 ± 0.06	31.44 ± 0.13	56.77 ± 1.08	6.76 ± 0.61	74.03 ± 2.50	90.98 ± 0.85	77.01 ± 1.86	44.66 ± 4.02	1079.5 ± 75.9

The range of residual ratios of total polyphenols was 77.01%–86.63% (Table 2, Figure 1C), and we found no effect of the cosolvent quantity on the residual ratio. In fact, the quantity of cosolvent seemed to exert less influence on the residual ratio of total polyphenols than it did on the removal ratio of caffeine. Regarding the effect of the quantity of initial water on phenolic fraction retention, Calvo *et al.* [42] showed that the no polyphenolic compounds were extracted from cocoa powder in samples treated at 30 MPa and 80 °C with 10% water addition. Under our experimental conditions, we were not able to completely prevent the disappearance of polyphenolic compounds. This difference would be caused from the higher quantity of SCCO_2_ used in this study compared to that in Calvo’s study.

On the other hand, the range of residual ratios of total procyanidins was 44.66%–85.58%. The residual ratio decreased remarkably as the quantity of cosolvent was increased (Table 2, Figure 1D). Interestingly, it appeared that procyanidins were extracted in greater proportions than other polyphenols, as the residual ratio of total polyphenols barely changed.

### 3.3. Effect of Temperature and Pressure on SCCO_2_ Extraction

**Figure 2:**
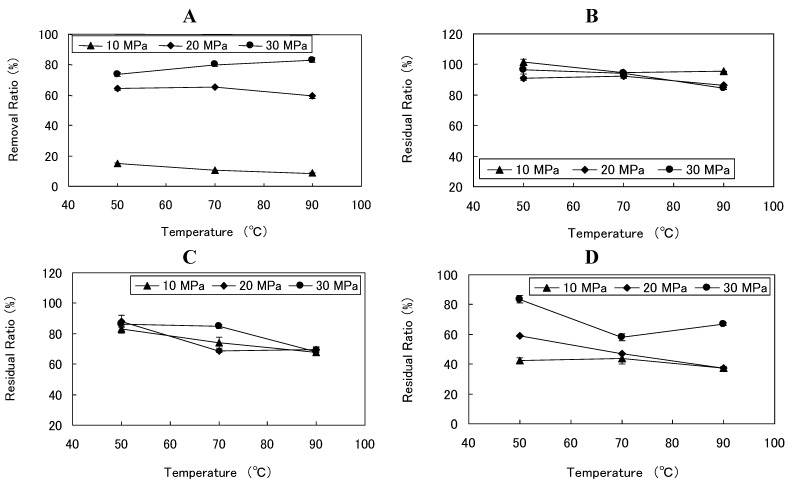
Effects of temperature and pressure on (**A**) caffeine removal ratio and (**B**) theobromine, (**C**) total polyphenols, and (**D**) total procyanidins residual ratios at a fixed cosolvent ratio of 45 wt% and a fixed S/F ratio of 55.6.

SCCO_2_ extraction was performed using nine different combinations of three different temperatures (50, 70, and 90 °C) and three different pressures (10, 20, and 30 MPa), at a fixed cosolvent quantity of 45 wt% in order to examine the influence of temperature and pressure on extraction. The removal ratio of caffeine rose slightly alongside temperature increases only at a pressure of 30 MPa. In addition, this ratio rose in response to pressure increases at each given temperature (Table 3, Figure 2A). This phenomenon would be caused by the solubility of caffeine in SCCO_2_ having increased with increasing temperature and pressure.

**Table 3:** Concentration and removal ratio of caffeine, concentration and residual ratio of theobromine, total polyphenols, and total procyanidins, and ORAC values in dry DCPs obtained by SCCO_2_ extraction at various pressures and temperatures.

SCCO_2_ Extraction Conditions			Concentration (mg/g)				Removal ratio (%)	Residual ratio (%)			ORAC (μmol-TE/g)
Pressure (MPa)	Temperature (°C)	Cosolvent (wt%)	Caffeine	Theobromine	Total polyphenols	Total procyanidins	Caffeine	Theobromine	Total polyphenols	Total procyanidins	
10	50	45	1.85 ± 0.01	29.99 ± 0.13	52.44 ± 2.45	6.43 ± 0.28	14.91 ± 0.69	101.41 ± 2.08	83.05 ± 2.54	42.25 ± 1.88	902.6 ± 12.5
10	70	45	2.03 ± 0.02	29.21 ± 0.23	48.73 ± 1.70	6.61 ± 0.53	10.55 ± 0.15	94.72 ± 0.34	74.10 ± 3.43	43.69 ± 3.52	911.6 ± 42.3
10	90	45	2.02 ± 0.02	28.70 ± 0.30	43.34 ± 1.08	5.64 ± 0.17	8.67 ± 0.39	95.45 ± 0.34	67.55 ± 1.24	37.22 ± 1.09	555.3 ± 21.2
20	50	45	0.86 ± 0.00	29.65 ± 0.55	61.19 ± 0.72	8.92 ± 0.07	64.19 ± 1.38	90.79 ± 1.31	87.92 ± 3.94	58.92 ± 0.46	1001.5 ± 59.6
20	70	45	0.90 ± 0.01	32.43 ± 0.28	51.39 ± 0.33	7.12 ± 0.06	65.41 ± 0.73	92.32 ± 1.23	68.57 ± 0.77	47.01 ± 0.39	1006.9 ± 55.9
20	90	45	0.94 ± 0.04	27.14 ± 0.20	46.73 ± 1.16	5.66 ± 0.02	59.56 ± 1.93	86.18 ± 0.49	69.55 ± 1.60	37.40 ± 0.13	874.5 ± 38.8
30	50	45	0.68 ± 0.02	33.75 ± 0.00	64.28 ± 1.09	12.65 ± 0.39	73.52 ± 1.47	96.66 ± 2.97	86.25 ± 1.19	83.56 ± 2.58	1164.2 ± 34.1
30	70	45	0.52 ± 0.03	33.44 ± 0.08	64.17 ± 0.98	8.79 ± 0.37	80.12 ± 1.05	94.12 ± 0.02	84.67 ± 1.13	58.06 ± 2.43	1218.5 ± 50.0
30	90	45	0.45 ± 0.02	30.15 ± 1.41	52.33 ± 0.05	10.11 ± 0.16	82.86 ± 1.53	84.29 ± 0.51	68.17 ± 2.87	66.77 ± 1.06	1057.1 ± 6.9

The residual ratio of theobromine was not greatly influenced by changes in pressure, but rose as temperature decreased. At a constant temperature of 90 °C, as pressure was lowered, the residual ratio of theobromine rose (Table 3, Figure 2B). As the solubility of theobromine in SCCO_2_ is known to be very low, there were reports of low ratio of theobromine extraction if conditions of a higher S/F ratio [43] or higher pressure along the lines of 80 MPa [44] are not met. Therefore, it was thought that the high residual ratio of theobromine in this study was caused by mild extraction conditions. 

The residual ratio of total polyphenols decreased in response to temperature increases at each given pressure. This ratio did not differ with changes in pressures at 50 °C and at 90 °C, but was greatly influenced by changes in pressure at 70 °C. This ratio reached a high at 70 °C, 30 MPa (Table 3, Figure 2C). In general, polyphenols cause thermal degradation at high temperature [45]. A rise in temperature reduces the density of the solvent but increases the vapor pressure of the solutes, causing thermal degradation. The lower residual ratio of total polyphenols at higher temperatures may be due not only to extraction by SCCO_2_ but also thermal degradation.

The influence exerted on the residual ratio of total procyanidins by temperature was different at each pressure (Table 3, Figure 2D). At 10 MPa, this residual ratio ranged from 37.22% to 43.69% across the three temperatures, and there were few influences on the ratio exerted by differences in temperature. At 20 MPa, the residual ratio decreased with temperature increases and reached a low of 37.40% at 90 °C. At 30 MPa, the residual ratio ranged from 58.06% to 83.56% across the three temperatures, and the residual ratio was lower at 70 °C than at 50 °C. These results show that the residual ratio of total procyanidins can be kept high under SCCO_2_ extraction if low temperature, high pressure conditions are maintained. The lower residual ratio of total procyanidins at higher temperatures may be due not only to extraction by SCCO_2_ but also thermal degradation. However, at 30 MPa, this ratio was higher at 90 °C than at 70 °C. In specific extraction conditions for a compound, the quantity of a certain ingredient extracted may decrease when the solubility of other ingredients specifically increases [46]. Similar phenomenon would be happened in our cocoa powder. In addition, our results showed that increase in pressure caused higher residual ratio of total procyanidins. This might be explained by our hypothesis that the solubility of other ingredients specifically increased with increasing pressure and were extracted with precedence over procyanidins, so that the residual ratio of total procyanidins might be high under high pressure conditions. 

An objective of the present study was to find the optimum conditions under which the desired DCP could be industrially manufactured. We regarded the length of the time as important factor in the industrialization in the extraction condition of pressure and temperature without fixing the S/F ratio in terms of time (kg-CO_2_/h/kg). Hence, increase in the S/F ratio in terms of time achieved at the lowest pressure (10 MPa) and the highest temperature (90 °C) was about 3.4 fold larger than that achieved at the highest pressure (30 MPa) and the lowest temperature (50 °C). Extraction time likewise greatly diminished where increase in the S/F ratio in term of time was higher. The extraction duration is the length of the time that the procyanidins are subjected to thermal degradation. Therefore, a difference in extraction duration might influence the residual ratio of total procyanidins. Furthermore, the removal ratio of caffeine might depend on the S/F ratio in terms of time.

In this study, using cocoa powder instead of cocoa beans [25] could raise the removal ratio of caffeine and promote efficiency of manufacturing cost and time. Furthermore, many functional ingredients such as theobromine and polyphenols could be retained in DCP.

### 3.4. Effect of SCCO_2_ Extraction Conditions on ORAC Value

**Figure 3:**
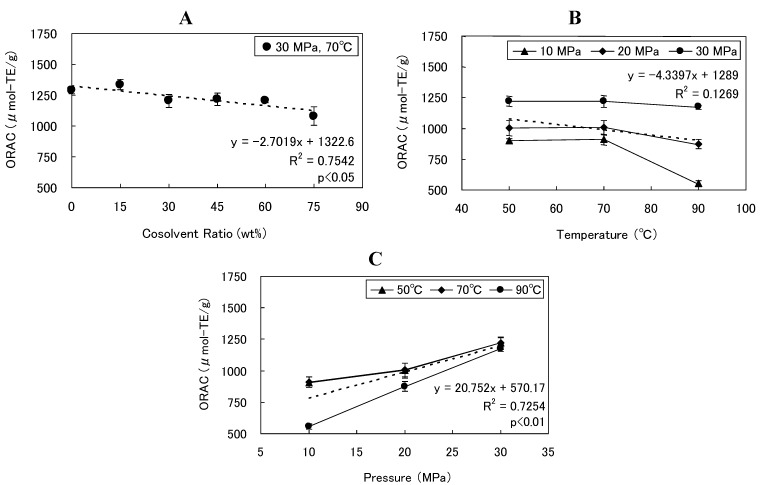
ORAC values observed after SCCO_2_ extraction with varying (**A**) cosolvent ratio, (**B**) temperature, and (**C**) pressure at a fixed S/F ratio of 55.6. Trend line is dashed.

**Figure 4:**
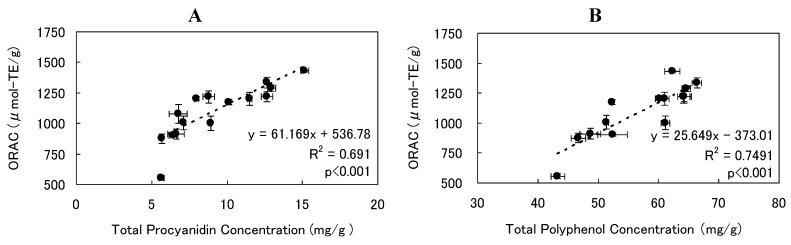
ORAC versus concentration of (**A**) total procyanidins and (**B**) total polyphenols in cocoa powder and DCP. Trend line is dashed.

We examined how SCCO_2_ extraction conditions, such as temperature, pressure, and cosolvent ratio were related to the ORAC value of DCPs. ORAC values ranged within 1079.5–1335.3 μmol-TE/g and a significant inverse correlation was observed between cosolvent ratio and ORAC values (*R^2^* = 0.75, *p* < 0.05, Figure 3A), but the influence of the cosolvent ratio on ORAC value was small in comparison with that of pressure. The ORAC value of DCP decreased with increasing temperatures, in particular during SCCO_2_ extraction trials at 10 MPa and 90 °C. However, significant correlation was not found between temperature and ORAC (Figure 3B). ORAC values could be kept high at higher pressure regardless of temperature, and a significant positive correlation was noted between pressure and the ORAC values of DCP (*R^2^* = 0.73, *p* < 0.01, Figure 3C). This result was due to an increase in the residual ratios of total polyphenols and total procyanidins as pressure increased. Furthermore, it has been reported that significant positive correlation was observed between total procyanidin concentration and ORAC for various cocoa powders and chocolate products [35,47]. We similarly found a positive correlation between total procyanidin concentration and ORAC values of untreated cocoa powder and DCP (*R^2^* = 0.69, *p* < 0.001, Figure 4A). In addition, it has been reported that quantity of total polyphenols and antioxidant ability decrease as fermentation progresses in cocoa beans sourced from Venezuela, and a relationship between total polyphenol quantity and antioxidant ability in cocoa beans has been shown [48]. Our results were in agreement, and we noted a positive correlation between the concentration of total polyphenols and ORAC values of DCP (*R^2^* = 0.75, *p* < 0.001, Figure 4B).

The correlation with ORAC was higher for total polyphenol concentration than for total procyanidin concentration. Therefore, the major factor determining ORAC of DCP would be some compounds in total polyphenols rather than total procyanidins. For example, there was no significant difference in ORAC between DCP made at cosolvent ratio of 0 wt% or 45 wt%. There was a significant difference in total procyanidin concentration, but total polyphenol concentration of both was almost the same value (Table 2). Based on the above, it was thought that a noticeable decrease in the DCP’s antioxidant activity might not occur even if total procyanidin concentration decreased substantially, as long as total polyphenol concentration could be kept at a certain level (Figure 1C,D and Figure 3A). Consequently, DCP retaining a relatively high total polyphenol concentration, such as 84.67% that of untreated cocoa powder, would retain antioxidant functionality. Further study would be necessary to identify other polyphenols besides procyanidins, which contribute to the antioxidant activity of cocoa powder.

## 4. Conclusions

We examined the conditions of SCCO_2_ extraction from cocoa powder. DCP with a caffeine content reduced by approximately 80%, and with theobromine content and polyphenol content retained at ratios higher than 94% and 84%, respectively, was obtainable by performing SCCO_2_ with a cosolvent ratio of 45 wt%, at 30 MPa, 70 °C, and the S/F ratio of 55.6. The antioxidant activity of this DCP was largely retained and observed to be 85.3% of the antioxidant activity of untreated cocoa powder. Because caffeine, which can have adverse effects on human health, was largely removed in this DCP without a great reduction in the quantity of theobromine or polyphenols, this DCP may have very few caffeine-linked adverse health effects. When dark chocolate is made with 40% untreated cocoa powder or 80% DCP, the caffeine content is 86.0 mg/100 g or 20.8 mg/100 g, respectively. Therefore, intake of conventional dark chocolate might unfortunately have caffeine-based adverse health effects. Using DCP, instead of untreated cocoa powder, to make dark chocolate could largely decrease these adverse effects and result in health benefits due to high polyphenol content, making DCP prepared by SCCO_2_ a promising food with beneficial health effects.
